# The stigmatisation of abortion: a qualitative analysis of print media in Great Britain in 2010

**DOI:** 10.1080/13691058.2014.937463

**Published:** 2014-08-13

**Authors:** Carrie Purcell, Shona Hilton, Lisa McDaid

**Affiliations:** ^a^ Centre for Population Health Sciences, University of Edinburgh, Edinburgh, UK; ^b^ MRC/CSO Social and Public Health Sciences Unit, University of Glasgow, Glasgow, UK

**Keywords:** abortion, stigma, media analysis, reproductive health, Great Britain

## Abstract

The media play a significant part in shaping public perceptions of health issues, and abortion attracts continued media interest. Detailed examination of media constructions of abortion may help to identify emerging public discourse. Qualitative content analysis was used to examine if and how the print media in contributes to the stigmatisation of abortion. Articles from seven British and five Scottish national newspapers from 2010 were analysed for overall framings of abortion and emergent themes, including potentially stigmatising discursive constructs and language. Abortion was found to be presented using predominantly negative language and discursive associations as ‘risky’, and in association with other ‘discredited’ social practices. Key perspectives were found to be absent or marginalised, including those of women who have sought abortion. Few articles framed abortion as a positive and legitimate choice. Negative media representations of abortion contribute to the stigmatisation of the procedure and of women who have it, and reflect a discrediting of women's reproductive decision-making. There is a need to challenge the notion that abortion stigma is inevitable, and to encourage positive framings of abortion in the media and other public discourse.

## Abstract (es)

Los medios de comunicación son especialmente importantes a la hora de formar las percepciones públicas en cuestiones de salud, y el aborto atrae continuamente el interés de los medios. Un examen detallado de las construcciones mediáticas del aborto podría ayudar a identificar los nuevos discursos públicos. Mediante un análisis cualitativo de contenido, examinamos si los medios impresos contribuyen a estigmatizar el aborto, y de qué forma. Se analizó la formulación general del aborto y temas emergentes, incluyendo el posible estigma mediante las construcciones discursivas y el lenguaje, de artículos de siete periódicos nacionales británicos y cinco escoceses de 2010. Se observó que el aborto se presentó: mediante el uso de lenguaje y asociaciones discursivas predominantemente negativos, como “arriesgado”, y vinculado a otras prácticas sociales “desacreditadas”. Se observó una falta o marginalización de perspectivas importantes, incluyendo las de las mujeres que querían abortar. En pocos artículos se presentaba el aborto como una opción positiva y legítima. Las representaciones negativas del aborto que se observan en los medios de comunicación contribuyen a estigmatizar esta práctica y a las mujeres que la utilizan, y reflejan la deslegitimación de las decisiones reproductivas de las mujeres. Es necesario cuestionar la noción de que el estigma del aborto es inevitable, y estimular la formulación positiva del aborto en los medios de comunicación y otros discursos públicos.

## Abstract (fr)

Les médias jouent un rôle essentiel dans la détermination des perceptions du public sur les questions de santé, parmi lesquelles l'avortement qui suscite un intérêt constant de leur part. Un examen détaillé des constructions médiatiques sur l'avortement peut aider à identifier le discours public qui en émane. L'analyse qualitative du contenu a été employée pour examiner si et comment la presse écrite contribue à la stigmatisation de l'avortement. Publiés en 2010, les articles de sept journaux britanniques et de cinq journaux écossais d'envergure nationale ont été analysés pour déterminer la formulation générale de l'avortement et des thèmes associés, y compris les constructions et le langage discursifs incitant potentiellement à la stigmatisation. Cette analyse a révélé que l'avortement était présenté: dans un langage négatif prédominant, accompagné d'associations discursives; comme « dangereux »; et comme associé à d'autres pratiques sociales « discréditées ». L'absence et la marginalisation des perspectives clé ont elles aussi été révélées, notamment les points de vue des femmes ayant souhaité interrompre leurs grossesses. Peu d'articles avaient présenté l'avortement comme un choix positif et légitime. Les représentations négatives des médias sur l'avortement contribuent à la stigmatisation de cette pratique et des femmes qui y ont recours. Elles sont le reflet du discrédit que subissent les prises de décision des femmes en matière de reproduction. La notion selon laquelle le stigma de l'avortement est inévitable doit être remise en question. De même, la formulation de l'avortement dans les médias et dans les autres discours publics, sous un angle plus positif, doit être encouragée.

## Introduction

The mass media play an integral part in shaping, or providing context for, public opinion (Gamson and Modigliani 1989). They constitute one sphere in which what is ‘normal’ or taken-for-granted is communicated on a large scale (Altheide and Schneider 2013), and messages about trust, fear, risk and blame are conveyed (Oaks 2003). Newspapers may offer a discursive space in which readers can identify and converse with an ‘imagined community’ (Anderson 1991; Seale 2003).

Detailed analysis of print media data enables us to consider the social context in which stories are produced, and to examine and understand the interaction between media representations and normative understandings/attitudes (Altheide and Schneider 2013). Interrogating how a health issue is constructed – the overall framing and specific language used – is crucial to understanding how it might be interpreted and experienced in everyday life. Examining media constructions of health issues is important for identifying emerging public discourse, and for understanding the ways in which phenomena might be normalised or stigmatised. This is crucial to understanding the impact they might have on not only the general public, but the healthcare providers and policymakers, by whom media representations are also consumed (Seale 2003).

A substantial amount of information on reproductive technologies is conveyed via the media (Ginsberg and Rapp 1995). These representations offer a public ‘evaluation of the reproductive choices’ women make, choices which may be negatively framed by the British media (Brown and Ferree 2005, 9). Where framed positively, they are undermined by negative associations, in a predominantly pronatalist discourse (Brown and Ferree 2005). Constructions of ‘appropriate’ timing of motherhood, and critical portrayals of older mothers in the British media have also been highlighted: Shaw and Giles (2009) suggest that negative language (labelling older mothers ‘irresponsible and selfish’) undermines the empowerment women might otherwise experience regarding reproductive ‘choice’. Despite the pronounced heteronormativity of the articles Shaw and Giles analysed, men were apportioned little responsibility for reproductive matters, a finding echoed in our own research (Martin, Hilton, and McDaid 2013).

Existing research on media representations and women's reproductive health primarily comes from the USA: for example, examining the ‘construction of the at-risk [female] reproductive body’ (Oaks 2003, 85); and the tendency of the US media to focus on abortion as a moral, religious and legal issue (Miller 1996). A notable shift has been identified in representations of abortion-related issues from a focus on women's reproductive rights and the risks of illegal abortions in the 1960s and 1970s, to the question of harm to the foetus from the 1980s onwards (Singer and Endreny 1987). A recent analysis of US film and television plots found abortion-related plots were relatively common, but an unrealistic emphasis on abortion-related mortality may reinforce moral and social myths associating abortion and death (Sisson and Kimport 2014). In a European context, links have been examined between the stigmatisation of abortion in the media, women's reproductive decisions, and nationalist discourse (Kirkham 2013). Media representations have been found to present the stigma of abortion as ‘a universal social fact’ (Kumar, Hessini, and Mitchell 2009, 627), rather than a socio-cultural construct. Together these illustrate ways in which the politics of reproduction and reproductive agency, and culturally-specific constructions of womanhood/femininity are reflected in media representations of abortion. However, as yet, no research has addressed the presentation of abortion in the British media specifically and in depth.

Abortion is a subject which is continually contested in the worldwide media. The cases of women who challenged Ireland's restrictive abortion laws in the European Court of Human Rights in 2010; and the death in 2012 of Savita Halappanavar (from septicaemia after being denied an abortion) have maintained the presence of abortion rights in the Republic of Ireland in news reporting. The case of ‘Beatriz’, a Salvadoran woman denied an abortion despite an unviable pregnancy and significant risk to her life, garnered international media attention; as have increasing restrictions on access to abortion in the USA. Whilst high profile cases make the headlines, it is worth noting that restricted access in many countries means there are an estimated 21.6 million unsafe abortions and 47,000 abortion-related deaths worldwide each year (World Health Organization 2011).

Abortion is the most commonly performed gynecological procedure in the UK (Kumar et al. 2004). Within Great Britain (Scotland, England and Wales) it is regulated by the Abortion Act 1967, and is legally available to term to save the life of the pregnant woman, where her physical or mental health is in grave danger; or for severe fetal anomaly. It is also available to 24 weeks, where there is ‘greater risk than if the pregnancy were terminated, of injury to the physical or mental health of the pregnant woman’ (the grounds under which the majority of abortions in Great Britain take place). We draw a distinction in this paper between Great Britain and the UK because the Abortion Act does not extend to Northern Ireland and given the very different legal and policy context of the latter, where abortion is only permitted within a narrow set of circumstances (though note that a morally conservative discourse on abortion has also been reported there, Bloomer and O'Dowd 2014). Current statistics suggest 11,777 abortions took place in Scotland and 185,331 in England and Wales in 2013; equating to rates of 11.2 and 15.9 abortions per thousand resident women respectively (Department of Health 2014; Information Services Division 2014).

In this paper we examine whether, and in what ways, British newsprint representations of abortion contribute to its stigmatisation. Following Goffman (1963), we understand stigma as a social process by which an individual or group becomes ‘discredited’; a process which involves labelling difference, stereotyping, othering and discrimination (Link and Phelan 2001). Drawing on this concept we unpack two key themes: stigma and framings of abortion; and marginalised perspectives and spaces for contestation of abortion stigma.

## Methods

We selected seven British and five Scottish national newspapers (including their Sunday counterparts) with high circulation figures (National Readership Survey 2014), representing three genres: ‘serious’, ‘mid-market’ and ‘tabloid’. We have used this typology elsewhere, and it represents a range of readership profiles diverse in terms of age, social class and political ideology (Hilton, Patterson, and Teyhan 2012; Hilton and Hunt 2011; Hilton et al. 2010). We did not include any Northern Ireland-specific newspapers in our sample given the different legal and policy context noted above.

Articles were identified using the electronic databases Nexis UK and Newsbank (for *The Sun*, *The Daily Mail* and *The Mail on Sunday*) for the time period 1 January to 1 December 2010. Broad search terms (e.g., sexual and health, abortion, termination, STI, safe and sex, teenage and pregnancy) were used to ensure a wide range of articles could be assessed for inclusion as part of a broader study of representations of sexual health issues in the British print media in 2010 (Martin, Hilton, and McDaid 2013). All articles were read by the first author (CP) to determine whether they met two inclusion criteria: that abortion was the main focus of the article, and that the article addressed the British context (since the study aimed to identify Britain-specific issues). CP conducted initial coding of all relevant text in NVivo 10, and this was refined in discussion amongst all authors. SH and LM then read a sub-sample of articles in order to develop a coding frame around the *a priori* research question: ‘In what ways, if any, might news reporting in Great Britain contribute to the stigmatisation of abortion?’ This question was designed to focus the analysis of a rich and varied data set on one key issue identified in the initial coding. Ninety-one articles met the inclusion criteria and were subsequently coded for further analysis by CP (Table 1).

**Table 1:** Summary of articles by genre (*n* = 91).

Genre	Newspaper title	No. of articles
‘Serious’	*The Telegraph* and *The Sunday Telegraph*	18
	*The Guardian* and *The Observer*	8
	*The Independent* and *The Independent on Sunday*	4
	*The Scotsman* and *Scotland on Sunday*	3
	*The Herald* and *Sunday Herald*	3
Mid-market	*The Daily Mail* and *The Mail on Sunday*	19
	*The Daily Express* and *Sunday Express*	12
	*Edinburgh Evening News*	2
Tabloid	*The Sun*	12
	*The Daily Mirror*	5
	*Daily Record*	5
	Total	91

Thematic analysis was then conducted manually to address the framing in each article as a whole, as well as commonalities and differences in language across the sample. A ‘constant comparative’ approach (Glaser and Strauss 1967; Lincoln and Guba 1985) was adopted to identify thematic patterns and develop explanations for differences. Articles were read for emergent themes such as negative language and discrediting associations.

From this descriptive stage, we moved onto a conceptual stage analysing emerging ‘latent’ content, including less explicit and perhaps unintended themes (Clarke and Everest 2006). Thematic categories were re-read paying attention to framing and language that might potentially stigmatise abortion. We also examined the more subtle or implicit meanings suggested by associations made in the articles; and how these relate to normative constructions of women, femininity and reproduction.

## Results

In-depth analysis of articles identified two key themes relating to British print media representations of abortion, which contribute to its stigmatisation, namely: the use of negative language and discursive associations; and the marginalisation of key perspectives such those presenting abortion as a positive and legitimate choice.

### Framings of abortion: negative language and discursive associations

A number of sub-themes emerged in relation to the language and associations made in the media framings of abortion. The theme of *controversy, sensationalism and morality* addresses the way in which language choices and sensationalist framings perpetuate abortion stigma, and normalise a moralising stance towards it. The *abortion risks* theme addresses the ways in which abortion is constructed both as emotionally and physically risky. *Stigmatising associations and oppositional tropes* examines the impact of discursive connections and contrasts made between abortion and other phenomena. Lastly, *women and femininity: constructions of an ‘other’* addresses implications of the language used to account for women having abortions, and how this is situated in relation to broader gender constructs.

#### Controversy, sensationalism and morality

One immediately striking feature of the language used in the media coverage we examined was that abortion was presented as unquestionably and perpetually ‘controversial’:

If one topic in medicine is guaranteed to generate controversy, it's abortion. The mere mention of it immediately polarises opinion. (*The Telegraph*, May 24, 2010)

The never-ending controversy over abortion may be about to enter a disturbing new phase. (*The Daily Mail*, November 3, 2010)

This positioned abortion as something unusual, atypical and which cannot be normalised, belying the fact that it is Great Britain's most often performed gynaecological procedure. By offering no alternative framings of abortion as unremarkable and commonplace, and by putting conflict centre-stage as a feature of public discourse on abortion, the controversy and stigma of abortion are perpetuated.

Many articles tended to rely on emotive language, with abortion and related issues presented as ‘flying in the face of morality’ (Society for the Protection of the Unborn Child [SPUC] quoted in *Mail*, October 10, 2010); and abortion statistics as painting ‘a profoundly depressing picture of modern Britain’ (Christian Medical Fellowship spokesperson quoted in *Sunday Telegraph*, June 13, 2010). Advertising for a post-conception advice line was described as ‘sick’, ‘grotesque’ and ‘tragic’:

To allow abortion providers to advertise on TV, as though they were no different from car companies or detergent manufacturers, is grotesque. (Life spokesperson quoted in *The Guardian*, May 20, 2010 and *The Independent*, May 22, 2010)

Much of this emotive and explicitly moralising language came from anti-abortion groups, and the pervasiveness of these was striking, albeit unsurprising. However, journalists and columnists also drew on tropes of (women's) irresponsibility and immorality, here in the context of another stigmatised reproductive practice:

EIGHTY women a year undergo costly IVF then have ABORTIONS – dozens just because they have second thoughts about being a mum. The shock figure sparked outrage yesterday that some women were callously treating test-tube babies like ‘designer goods’. (*The Sun*, June 7, 2010. Block capitals in original text)

The focus on numbers implies that there is somehow a ‘correct’ number for abortions, and that 80 is too high. It also obfuscates the numerous reasons why termination might be sought following IVF and skews the story away from medical grounds, including fetal anomaly: an issue which The Human Fertilisation and Embryology Authority (the UK's IVF regulator) responded to in a statement (Human Fertility and Embryology Authority 2011). By reducing the issue to a woman ‘having second thoughts’, it fails to recognise the complexities and stresses of assisted conception, which may contribute to a termination being sought, or the likely emotional difficulty of the decision. Instead it dichotomises pregnancy by positioning IVF pregnancies as *planned/wanted* and aborted pregnancies as *unplanned/unwanted*. While this example typifies the ‘tabloid’ style, it also illustrates many of the narrow assumptions underpinning presentations of abortion across the sample.

#### Abortion risks

The data included explicit discourse of risk associated with abortion: ‘Abortion triples breast cancer risk’ (*The Daily Mail*, June 24, 2010). Of equal interest were the more subtle linguistic framings in which abortion was presented as an unacknowledged risk to women:

Margaret Forester passed the booklet to family planning staff at the health centre where she worked because she felt that the NHS was not offering patients enough information about the risks associated with terminating a pregnancy. (*The Telegraph*, December 22, 2010)

Others complained that [an advert for post-conception advice] misled viewers by not referring to the physical and mental health risks of abortion and failed to mention that pregnant women who wanted advice could contact their GPs. (*The Herald*, August 4, 2010)

Use of the definite article here might be seen to subtly imply that these claims are accepted and inarguable, rather than highly contested. Phrases stating that abortion providers were ‘failing to warn [women] that what they are doing to their bodies – the quick fix of abortion – can do grave harm’ (*The Daily Mail*, June 24, 2010) can be interpreted in different ways. On the one hand, they may imply that genuine scientific evidence is being withheld from women and/or that they are being misinformed (a charge that existing research on the dissemination of abortion information has levied at the US media itself, Miller 1996). In this sense it can be aligned with either a discourse of mistrust of the medical establishment; or with a deliberate attempt by those in favour of abortion to mislead women. However, this phrasing can also be read as putting the responsibility for abortion squarely at the feet of women (‘what they are doing to their bodies’), with the caveat that they may not fully comprehend the impact of their action. On the one hand women were viewed as having little or no agency to make informed decisions; while on the other, any decisions they did make were characterised as flawed.

One anti-abortion campaigner claimed women had varied responses to abortion, which were equally negative:

There are two common reactions to abortions – either to have an abortion, deeply regret it and vow never to have another, or to have an abortion and feel numbed from emotion. (Care Confidential spokesperson quoted in *The Daily Mirror*, February 18, 2010)

An ‘emotional risk’ framing – in which abortion could be the source of ‘guilt, soul searching, and the colossal potential for regret’ (*The Daily Express,* August 10, 2010) extending throughout the rest of the woman's life – preyed not only on general anxieties about the unknown and the desire to avoid stigmatisation, but also specifically on women's anxieties about future childbearing and happiness. The repeated suggestion that abortion presented a physical or psychological *danger* to women is nonetheless at odds with scientific evidence.

A further point regarding this discourse of risk is the framing of abortion as something that should be concealed. It is thus risky in the sense that it might be exposed. This was evident in articles relating to two teenagers whose ‘abortion secret’ had come to light after an NHS nurse reportedly informed their families (*The Sun*, January 29, 2010; *The Sun*, February 1, 2010; *The Telegraph*, January 29, 2010). The young women's reasons for seeking an abortion, or for keeping it secret (other than passing reference to one's ‘Catholic’ family), were given scant attention. The emphasis instead centred on their distress at having been ‘disowned’ and ‘thrown out of home’. One inference is that, regardless of your reasons (or your right to privacy or confidentiality), having an abortion is a negative attribute which, if discovered, could impact on your life and relationships.

#### Stigmatising associations and oppositional tropes

The language used to construct abortion fluctuated between framing it as an individual problem and a social failing, often conveyed through associations with other stigmatised phenomena. Abortion was aligned with widely discredited, ‘deviant’ and/or sometimes unlawful practices, which speaks to a discourse of moral panic (see Table 2 for examples). Rather than locating it in a positively-framed spectrum of women's reproductive practices or life choices, these framings magnified stigma and controversy by aligning abortion with readers' (assumed to be negative) pre-existing views.

**Table 2:** Examples of stigmatising associations between abortion and other phenomena.

Association: abortion and…	Quotation
Immigration/health tourism	‘Every year thousands of Polish “abortion tourists” travel to Britain where they can have the procedure for free under EU regulations. As long as they can claim the termination is an “emergency”, they do not have to pay.’ (*The Daily Mail* 16 March 2010)
Binge drinking	‘The devastating impact binge drinking has on young women has been exposed in a shocking study. The report found that “ladettes” who drink to excess are 40 per cent more likely to have an abortion.’ (*The Daily Express*, 21 August 2010)
Terrorism and slavery	‘Lord Nicholas […] claims that abortion is a bigger threat to Europe than al-Qaeda and Islamic terrorism. He describes abortion as “the single most grievous moral deficit in contemporary life” and calls for a “new abolitionism for Europe” in which abortion, like the slave trade, can be abolished.’ (*The Telegraph*, 20 December 2010)
Underage sex/teen pregnancy	‘The number of schoolgirls having abortions has reached a five-year high in the Lothians. New figures have revealed there were 108 underage pregnancies in the region last year, with 71 being terminated.’ (*Edinburgh Evening News*, 30 June 2010)
Promiscuity/ casual sex	‘There were concerns it [post-conception advice advert] could encourage women to have an abortion, when they had not previously considered the option, and could encourage promiscuity.’ (*The Herald*, 4 August 2010)
Rape	‘“I am pro-choice. But I am not pro-choice about rape, burglary, kidnapping or killing children”.’ (*The Independent*, 26 October 2010)

Oppositional tropes were another linguistic tool used in the articles. General practitioners were termed ‘family doctors’ (*The Telegraph*, April 16, 2010; *The Daily Express*, April 16, 2010); and those citing objections to abortion – namely the various faith-based groups, and conservative pressure-group Mediawatch – were euphemistically called ‘family campaigners’ (*The Daily Mail*, February 23, 2010; *The Daily Express*, May 21, 2010; *The Sunday Express*, June 6, 2010). Abortion was therefore contrasted with notions of ‘family’, implying their mutual exclusivity or incompatibility. Interestingly, beyond general reference to organisations like British Pregnancy Advisory Service (BPAS), little reference was made to staff that actually provide abortion services. Scant references appeared to ‘abortion doctors’ (an equally stigmatising term), and these were most common in relation to the US context.

Abortion and motherhood were presented in oppositional terms in relation to the suggestion that abortion causes breast cancer:

A team of scientists made the claim while carrying out research into how breast-feeding can protect women from developing the killer dis-ease [sic]. While concluding that breast-feeding offered significant protection from cancer, they also noted that the highest reported risk factor in developing the dis-ease [sic] was abortion. (*The Daily Mail*, June 24, 2010)

Abortion and breast cancer are ‘highly visible, politicised, and emotionally laden women's health issue[s]’ (Oaks 2003, 79); and one reading of this comment is that the contrast between breast-feeding (good) with abortion (bad) maps onto a juxtaposition of compliance with, and rejection of, the ‘feminine idea’ of motherhood (Kumar, Hessini, and Mitchell 2009). The norm is underlined by the implication that its transgression could put a woman's health in danger. In vitro fertilisation (IVF) and abortion were similarly polarised – with abortion *following* successful IVF presented as ‘beyond the pale’ (*The Daily Express*, June 6, 2010; *The Daily Express*, June 8, 2010), rather than as technological interventions representing different aspects of women's reproductive choice. Women seeking abortion following assisted conception were implied to be indecisive and capricious.

#### Women and femininity: constructing an ‘other’

It is useful to consider the discursive constructions of women and femininity, and the ways in which these construct women who have abortions as a distinct group, somehow different to ‘normal’ women. Broadly speaking, women who have abortions were typified (particularly in the shorthand of headlines) as childless; perhaps single, rather than part of existing ‘families’ (as discussed above); and as teenagers or ‘girls’, rather than older women:

‘Four abortions … Now I'll never have children’; With the number of women having multiple abortions rising in Britain, Karen Collier explains why she's had four – and her devastation at now being left childless. (*The Daily Mirror*, February 18, 2010)

‘At 19, Sarah had an abortion. Now 38 and childless, an email from her unborn baby's father has made her question her whole life.’ (*The Daily Mail*, April 16, 2010)

Whilst abortion rates tend to be highest amongst women in their late teens and early 20s (Department of Health 2014; Information Services Division 2014), the tendency of reporting to focus almost *exclusively* on this group plays into moral panics around teenage pregnancies, and silences the many women terminating pregnancies in their 30s and 40s. It also serves to marginalise those who do so when they already have children, concern for whose well-being may be foremost in their decision to abort.

The testimonies of women who have actually had abortions went some way to counter these typifications, in that a number of those presented had children, were in established relationships, married, or older. These numbered only six articles in a sample of 91, however, and overall the personal testimonies contributed to negative framings in that they presented the decision to terminate as one made easily by a younger woman, but as one which an older woman was likely to regret. Testimonies from women in their late 30s to 50s presented mixed or negative accounts of the aftermath of abortions. Stories highlighted women's (by implication erroneous) assumption that they had ‘plenty of time’ to meet a partner and create the ideal circumstances in which to have children, the implication being that if you abort, you may miss your only chance to have a child. Such framings are troubling for their punitive tone and suggestion that not having children is the ‘ultimate price’ that women might pay for anything. They are also problematic for the fact that they were not countered by any alternative presentations of women who do not want to have children at all, or who had abortions followed by, or in the midst of, a happy and healthy family life.

The implication that women who have abortions belonged to a particular ‘type’ is also highly problematic. Firstly it gives a reductionist view of the reasons why women might want or need an abortion. Secondly, it denies the complexity of a situation in which any woman might find herself: that is, faced with a pregnancy with which she feels unable to cope. Thirdly, it is also recognisable as part of a process of othering in which ‘women who have abortions’ are set apart from the rest of society, associated with an undesirable characteristic, and thus stigmatised.

The specific negative terms in which women who have abortions were described framed them as ‘irresponsible’; ‘immoral’; ‘incapable’ (of looking after themselves, of managing contraception or their own sexuality); ‘selfish’; as not behaving ‘respectably’; and as ‘pathetic’ and ‘wretched’. Similarly to other health contexts, media discourse around abortion positions women in a complex and problematic way, as being primarily responsible for any harm they may come to, but simultaneously vulnerable and in need of protection (Miller 1996). Some articles suggested women needing abortions might be pitied, with one labelling them ‘poor, frantic females’ (*The Sun,* May 22, 2010): a phrase that undermines the rationality with which women might decide to abort. Sympathy was most evident in the language and tone of accounts of women ending their pregnancies on health grounds, who had been ‘happily pregnant’ but then became ‘uncontrollably ill’ (*The Sun,* September 23, 2010). In this context women were accounted for as having ‘suffered in silence’ (*The Sun*, September 23, 2010), and as having been through a traumatic ‘ordeal’ (*The Sun,* June 25, 2010), which justifies their decision to abort. A physiological justification for termination appeared to be markedly more acceptable in news discourse than one grounded in mental health, emotional or social factors.

Furthermore, personal testimonies suggested that women expected to be stigmatised for seeking abortion. Phrases such as ‘[o]bviously I'm not proud of what I've done and I know people will judge me’ (*The Sun,* November 10, 2010) served to reinforce and normalise moralising discourse and underscore abortion stigma, by focusing on negative emotions like guilt, disgust, trauma and shame. Whether this is evidence of the internalisation of stigma by women who have had abortions, or of the deliberate selection of women who have had particularly negative experiences by the articles in question, is impossible to say. It is possible, however, that women giving accounts of abortion may feel the impact of social desirability bias and feel obliged to speak in negative terms, citing their feelings of ‘shame’, ‘heartbreak’ and ‘regret’. Moreover, women may describe abortion as a ‘horrendous decision’ – which ‘no woman wants to have to make’, and which is ‘the hardest decision in the world’ – because these are the dominant tropes available to them with which to account for their experience.

### Marginalised perspectives and minor contestations

We found that a number of perspectives were notable by their absence or marginalisation in the data, principally: the framings of abortion as a positive or legitimate choice; the voices of women; the role of men; and contestations of abortion stigma.

#### Abortion as a positive and legitimate choice

A minority of positive framings did appear, where, for example, one woman stated that the decision was ‘fixing’ something and the ‘right thing to do’ (*The Guardian*, November 13, 2010). Another woman who had three abortions stated that she ‘didn't feel guilt about the terminations, which to me were medical procedures’ (*The Scotsman,* February 9, 2010). However, such positive statements were commonly followed by a significant counterpoint. The latter article immediately went on to say ‘[m]y friends were disgusted with me and asked how long I was going to carry on like that’. This effectively undermined her assertion since, whilst she may not have had negative feelings regarding the abortions, those around her did.

Women's reasons for seeking abortion were in some instances dismissed as irrelevant because the procedure was assumed to be so ‘traumatic and terrifying’ as to apparently outweigh any rationale in its favour (*The Daily Mirror*, February 18, 2010). Where it was presented as a choice, it was a painful one:

‘I was sick 40 times a day, shaking and too weak to walk – until it got so bad I had an abortion.’ Women tell how their morning sickness led to tragic choice. (*The Sun,* September 23, 2010)

But, in truth, for most women, termination is an agonising choice and not one taken lightly. (*Daily Record*, May 21, 2010)

Abortion was also presented as a ‘lifestyle choice’:

But for many, abortion appears to have become alarmingly casual, a form of birth control, a lifestyle choice, a minor medical procedure to be booked for a day off. (*The Daily Mail*, November 3, 2010)

The phrase ‘lifestyle choice’, alongside the suggestion that women used abortion as an alternative to contraception, could be taken to imply a superficial or poorly considered decision, rather than a practical response to an unintended or unviable pregnancy. Moreover, this characterisation of abortion marks the set of values associated with this supposed ‘lifestyle choice’ as undesirable and objectionable: another key step in the stigmatisation process.

#### Absent men and invisible women

The men who co-conceived the pregnancies being aborted rarely featured. Only one article addressed this absence, particularly around questions of responsibility. It pinpointed and criticises the ‘repulsive’ normative attitude that ‘girls “get into trouble”, while boys have “healthy urges” they can't control’ (*The Telegraph*, June 14, 2010), and is notable in its uniqueness. As our analysis highlights, women who have actually sought abortion were also conspicuously absent. Particular topic areas – such as the concern with gestational time limits or foetal pain – prioritise concerns for the foetus, and see women discursively erased, receiving little or no mention in articles covering these issues. With less than 10% of our overall sample addressing women's own accounts of abortion, lived experience took a back seat to moral judgements and generalised claims about abortion.

#### Contesting abortion stigma

The above examples are offset by just one instance in which an article suggested abortion (here in the context of foetal anomaly) might be considered positively as a ‘moral choice’:

…the fact that nobody will talk about [abortion] as a moral choice means that the act becomes more and more shameful, as the silence reinforces itself. (*The Guardian,* October 5, 2010)

The same article suggested that the apparent dominance of anti-choice positions in the British media have arisen because ‘when the pro-choice movement won the legislative argument, it ceded the moral high ground as a consolation prize to the anti-abortionists’ (*The Guardian* October 5, 2010). Whether or not this is so, that only a small minority of articles created any discursive space in which abortion could be framed positively suggests that anti-abortion arguments could be privileged. Some explicitly pro-choice perspectives were present in the data; however, these frequently came later in the article and were often followed by an undermining ‘but’ /or further comment from an anti-choice perspective.

One further article presented the lesser-heard argument that while abortion may well be difficult, it was chosen because the woman ‘takes motherhood seriously’ (Abortion Rights spokesperson quoted in *The Sun* November 10, 2010) and was taking responsible action in this respect. On the whole, the lack of focus on counter-narratives in which women's experiences were framed positively creates a dominant impression that abortion is an undesirable practice, with discrediting implications for anyone associated with it.

## Discussion

We conducted a qualitative analysis of representations of abortion in the British print media in 2010 to examine in what ways, if any, print news reporting might contribute to the stigmatisation of abortion in Great Britain. Stigma is a social process by which people become ‘discredited’ by association with a non-normative condition or practice (Goffman 1963). In specific relation to abortion, stigma can be conceptualised as ‘a negative attribute ascribed to women who seek to terminate a pregnancy that marks them […] as inferior to ideals of womanhood’ (Kumar, Hessini, and Mitchell 2009, 628). Such ideals are highly context-specific, and relate largely to notions of ‘perpetual fecundity’ and the ‘inevitability of motherhood’ (Kumar, Hessini, and Mitchell 2009, 625).

Abortion stigma overlaps with stigma associated with poverty, young motherhood/teen pregnancy and various forms of social exclusion, and may shape abortion provision. The ways in which abortion care providers typify women seeking abortion (by age, class etc.) may offer an example of ‘stratified reproduction’ (Beynon-Jones 2013, 509), that is, the process by which women's reproductive outcomes are stigmatised, or normalised (Ginsberg and Rapp 1995). It is therefore essential to address the potential for stigmatisation to result in discrimination against already disadvantaged groups (Kumar 2013).

Whilst we did find some small spaces for contestation and resistance, abortion was for the most part subject to consistently negative framing in the British print media in 2010. Associations with controversy, sensationalism and (im)morality marked abortion, and women who have abortions, as different and distinct. Associations with ‘deviant’ or discredited practices (teen pregnancy, binge drinking) and undesirable characteristics (promiscuity, selfishness) stereotype and falsely distance women who have abortions from the rest of society, in a way which is typical of the process of stigmatisation (Link and Phelan 2001).

Abortion stigma also relates to reproductive norms of ‘family planning’ and ‘appropriate’ forms of childbearing. Echoing existing research (Kumar, Hessini, and Mitchell 2009), our data presents abortion as incompatible with notions of the family, femininity and motherhood. Women who have abortions transgress a boundary and (whether intentionally or not) the representations we examined perpetuate reproductive norms by presenting such women as unhappy or unfulfilled. Furthermore, this stigmatisation relates to the perceived failure of women to manage their sexuality within tightly proscribed limits: namely bearing children when neither too young nor too old, whilst in a stable relationship and financially secure position. Media constructions of the appropriate or acceptable context for pregnancy, motherhood and abortion, are further evidence of the stratification of reproduction, whereby some women's reproductive ‘choices’ are stigmatised. In this sense, abortion stigma can be seen as having a regulatory role, discrediting women who behave in a way which does not fit with normative femininity. Elsewhere, it has been suggested that even positive discourses around equality, support and rights for abortion can be used by men to undermine women's reproductive rights (Macleod and Hansjee 2013).

Abortion stigma may be discursively enacted in various ways, such as implying that abortion is ‘dirty or unhealthy’ (Norris et al. 2011, S52). The specific notion of a ‘post-abortion syndrome’ – which attempts to medicalise supposed physical and psychological risks of abortion (see Dadlez and Andrews 2010; Steinberg and Finer 2011) – appears to have less of a foothold in our sample than in US abortion discourse. However, framings of abortion as physically, emotionally and socially ‘risky’ do appear throughout our sample. What it striking is that medically recognised risks of abortion – including high rates of maternal death where it is not provided *safely* (World Health Organization 2011) – go unacknowledged. Instead, abortion in Great Britain – where it *is* safely and legally provided – is portrayed as potentially dangerous.

It was also common in our sample for abortion to be presented as a risk to women's mental well-being. However, research suggests that there is no greater association between abortion and negative metal health outcomes than between childbirth and the same (Kero, Hogberg, and Lalos 2004). Conversely, stigma has been found to impact women's well-being following abortion, and raises concerns about whether (and with whom) they feel able to discuss their experience (Major and Gramzow 1999; Shellenberg et al. 2011). Key factors in negative psychological outcomes after abortion include concerns about ‘judgment’, ‘isolation’ and ‘community condemnation’ (Cockrill et al. 2013, 79); and a ‘need for secrecy’ and an expectation of low ‘social support’ for their decision (Major et al. 2009, 882). Taking together the potential for print media representations to shape public opinion, and the fact that many of the representations we analysed perpetuated factors associated with these negative outcomes, it can be argued that the British print media play a significant part in magnifying the difficulties some women may experience around abortion. Representations of abortion as non-normative and potentially dangerous not only contribute to stigma but could lead media consumers to believe general attitudes to abortion in Great Britain are negative, when research has found public opinion to be more generally in favour of reproductive choice. For example, a 2008 Ipsos MORI survey found 61% of women respondents of childbearing age believed women should have access to abortion for a range of medical and social reasons.

It should be acknowledged that journalistic practices lead to news being constructed in particular ways. Some justifications for abortion (extreme morning sickness) may be found to be more acceptable than others (non-use of contraception). This effectively creates a dichotomy of ‘good’ and ‘bad’ abortion (Rapp 2000); precisely the kind of narrative device upon which journalists commonly capitalise. Similarly, the use of associations as a means of discursive shorthand, and an emphasis on risk and controversy, are effective story-telling techniques, and their use in the context of abortion parallels findings on cancer stories (Clarke and Everest 2006).

Journalistic practices do not, however, contribute to explaining the media focus on ‘evaluating’ women's reproductive choices, which our analysis highlights. This predominantly negative evaluation emphasises ‘bad choices’ around sexual practices, in that women are held to account for needing abortions, and for the ‘choices’ that have got them to that point. This parallels earlier research on both abortion stigma and media representations of women's reproductive decisions (Brown and Ferree 2005; Norris et al. 2011), and is an evaluation which is not applied to men. References to the part played by men in reproductive decisions were almost entirely absent from the media representations we analysed, which implies that the responsibility for reproductive decisions lies almost exclusively with women (see Martin, Hilton, and McDaid 2013). However, references to women were made largely without giving the women concerned an active voice, which is suggestive of the broader effacement of women from the reproductive process by patriarchal institutions (Ginsberg and Rapp 1995). The absence of the perspectives of women who have sought abortion leads to narrow and reductive representations of abortion, which over-simplify the complexities of women's experience, and limit the discursive options available to women for interpreting their experience as a legitimate or positive one. Instead, representations tend to implicitly frame the debate around whether a woman should be blamed for an unintended pregnancy, and/or whether her justifications for seeking abortion are adequate, rather than whether or not she should have autonomy over her own body and reproductive decisions. With extremely limited representations of abortion as a legitimate or positive choice, regardless of women's reasons, we found no sustained print news discourse presenting abortion in a positive light.

### Study limitations

The data presented here are taken from one calendar year, and it is possible that a longer time frame would have allowed for the identification of more diverse representations of abortion (although the relative consistency in the negative language used across the sample suggests this may not be the case). Our analysis included only print news and therefore cannot be taken as representative of the wider media's role in representations and the stigmatisation of abortion.

## Conclusions

We found predominant framings of abortion to be negative, and abortion to be constructed as being at odds with norms of femininity. Voices of women who have sought abortions were marginalised, with women's reasons for seeking abortion at worst disregarded, and at best over-simplified. A discourse of abortion as a legitimate choice was largely absent. Since news media play a part in (re)producing social norms, it is useful to reflect on the impact of negative representations of abortion in the context of normative constructions of women and reproductive agency.

Ultimately, abortion stigma is pertinent for the impact it may have on access to services. Thousands of women in Great Britain every year, and millions worldwide, exercise their reproductive agency and have abortions. This may suggest a significant amount of resistance to stigmatisation (Kumar, Hessini, and Mitchell 2009). Nonetheless, other women may be reluctant to seek a service marked by stigma, and thus have their ability to exercise their reproductive rights constrained. Moreover, the millions of women who have abortions are likely to feel the impact of stigma, in whatever way it manifests locally, in the course of their abortion experience. There is therefore a pressing need to challenge the notion that abortion stigma is inevitable, and to encourage positive framings of abortion in the print media and other forms of public discourse.

## References

[cit0001] (2013). Qualitative Media Analysis.

[cit0002] (1991). Imagined Communities: Reflections on the Origin and Spread of Nationalism.

[cit0003] (2013). Expecting Motherhood? Stratifying Reproduction in 21st-Century Scottish Abortion Practice. Sociology.

[cit0004] (2014). Restricted Access to Abortion in the Republic of Ireland and Northern Ireland: Exploring Abortion Tourism and Barriers to Legal Reform. Culture, Health & Sexuality.

[cit0005] (2005). Close Your Eyes and Think of England: Pronatalism in the British Print Media. Gender and Society.

[cit0006] (2006). Cancer in the Mass Print Media: Fear, Uncertainty and the Medical Model. Social Science & Medicine.

[cit0007] (2013). The Stigma of Having an Abortion: Development of a Scale and Characteristics of Women Experiencing Abortion Stigma. Perspectives on Sexual and Reproductive Health.

[cit0008] (2010). Post-abortion Syndrome: Creating an Affliction. Bioethics.

[cit0009] Department of Health (2014). Abortion Statistics, England and Wales: 2013. https://www.gov.uk/government/publications/report-on-abortion-statistics-in-england-and-wales-for-2013.

[cit0010] (1989). Media Discourse and Public Opinion on Nuclear Power: A Constructionist Approach. American Journal of Sociology.

[cit0011] (1995). Conceiving the New World Order: The Global Politics of Reproduction.

[cit0012] (1967). The Discovery of Grounded Theory: Strategies for Qualitative Research.

[cit0013] (1963). Stigma: Notes on the Management of Spoiled Identity.

[cit0015] (2011). UK Newspapers' Representations of the 2009–10 Outbreak of Swine Flu: One Health Scare Not Over-hyped by the Media?. Journal of Epidemiology and Community Health.

[cit0016] (2010). Newsprint Media Representations of the Introduction of the HPV Vaccination Programme for Cervical Cancer Prevention in the UK (2005–2008). Social Science & Medicine.

[cit0014] (2012). Escalating Coverage of Obesity in UK Newspapers: The Evolution and Framing of the ‘Obesity Epidemic’ from 1996 to 2010. Obesity.

[cit0017] Human Fertility and Embryology Authority (2011). Statement on Daily Express Article on IVF Terminations.

[cit0018] Information Services Division (2014). Abortion Statistics: Year Ending 31 December 2013.

[cit0019] (2004). Wellbeing and Mental Growth—Long-Term Effects of Legal Abortion. Social Science & Medicine.

[cit0020] (2013). Constructions of Childhood, Victimhood and Abortion in Romania: The ‘Little-Girl Mother’. The Anthropology of East Europe Review.

[cit0021] (2013). Everything is not Abortion Stigma. Women's Health Issues.

[cit0023] (2004). Decision Making and Referral Prior to Abortion: A Qualitative Study of Women's Experiences. Journal of Family Planning Reproductive Health Care.

[cit0022] (2009). Conceptualising Abortion Stigma. Culture, Health & Sexuality.

[cit0024] (1985). Naturalistic Inquiry.

[cit0025] (2001). Conceptualizing Stigma. Annual Review of Sociology.

[cit0026] (2013). Men and Talk about Legal Abortion in South Africa: Equality, Support and Rights Discourses Undermining Reproductive ‘Choice’. Culture, Health & Sexuality.

[cit0027] (2009). Abortion and Mental Health: Evaluating the Evidence. American Psychologist.

[cit0028] (1999). Abortion as Stigma: Cognitive and Emotional Implications of Concealment. Journal of Personality and Social Psychology.

[cit0029] (2013). United Kingdom Newsprint Media Reporting on Sexual Health and Blood-borne Viruses in 2010. Sexual Health.

[cit0030] (1996). A Matter of Consequence: Abortion Rhetoric and Media Messages.

[cit0031] National Readership Survey (2014). RS Readership Estimates Newspapers and Supplements AIR Latest 12 Months: April 2013–March 2014. http://www.nrs.co.uk/downloads/pdf/newspapers_201401.pdf.

[cit0032] (2011). Abortion Stigma: A Reconceptualization of Constituents, Causes, and Consequences. Women's Health Issues.

[cit0033] (2003). The Politics of Health Risk Warnings: Social Movements and Controversy Over the Link Between Abortion and Breast Cancer.

[cit0034] (2000). Testing Women, Testing the Fetus: The Social Impact of Amniocentesis in America.

[cit0035] (2003). Health and Media: An Overview. Sociology of Health and Illness.

[cit0036] (2009). Motherhood on Ice? A Media Framing Analysis of Older Mothers in the UK News. Psychology and Health.

[cit0037] (2011). Social Stigma and Disclosure about Induced Abortion: Results from an Exploratory Study. Global Public Health.

[cit0040] (1987). Reporting Hazards: Their Benefits and Costs. Journal of Communication.

[cit0038] (2014). Telling Stories About Abortion: Abortion-Related Plots in American Film and Television, 1916–2013. Contraception.

[cit0039] (2011). Examining the Association of Abortion History and Current Mental Health: A Reanalysis of the National Comorbidity Survey Using a Common-Risk-Factors Model. Social Science & Medicine.

[cit0041] World Health Organization (2011). Unsafe Abortion: Global and Regional Estimates of the Incidence of Unsafe Abortion and Associated Mortality in 2008.

